# A randomized placebo-controlled double-blind study of dexmedetomidine on postoperative sleep quality in patients with endoscopic sinus surgery

**DOI:** 10.1186/s12871-022-01711-8

**Published:** 2022-06-01

**Authors:** Yu Wu, Yuhua Miao, Xuzhen Chen, Xiaojian Wan

**Affiliations:** 1 Department of Anesthesiology, Bethune International Peace Hospital, Shijiazhuang, 050082 China; 2 Department of Otorhinolaryngology, Bethune International Peace Hospital, Shijiazhuang, 050082 China; 3 Department of Anesthesiology and Critical Care Medicine, Changhai Hospital, Naval Medical University, Shanghai, 200433 China

**Keywords:** Endoscopic sinus surgery, Dexmedetomidine, Sleep quality

## Abstract

**Background:**

Postoperative sleep disorder is common and may cause aggravated postoperative pain, delirium, and poor prognosis. We accessed the effect of intraoperative intravenous dexmedetomidine on postoperative sleep quality in patients with endoscopic sinus surgery.

**Methods:**

This single-center, double-blind, placebo-controlled randomized clinical trial enrolled a total of 110 participants aged 18 years to 65 years who were scheduled to receive endoscopic sinus surgery. Placebo (normal saline) or dexmedetomidine infusion (load dose 0.5 μg kg^−1^ over 10 min, followed by maintenance dose 0.2 ug kg^−1^ h^−1^) during surgery. The primary outcome was postoperative sleep quality. Secondary outcomes were postoperative Ramsay sedation scores, Visual Analog Scale (VAS) scores, serum cortisol, 5-hydroxytryptamine (5-HT) and hypocretin, delirium, and postoperative nausea and vomiting (PONV).

**Results:**

Among enrolled 110 patients, 55 were randomized to administer intraoperative dexmedetomidine and placebo. In total, 14 patients (7 in each group) were excluded because of protocol deviations, and 96 patients (48 in each group) were included in the per-protocol analysis. The dexmedetomidine group had a significantly higher sleep efficiency index(SEI) (66.85[3.00] *vs* 65.38[3.58]), the ratio of rapid eye movement sleep to total sleep(REM)(13.63[1.45] *vs* 12.38[2.11]) and lower arousal index (AI) (7.20[1.00] *vs* 8.07[1.29]), higher Ramsay sedation score at post-operation 1 h, 12 h point, lower VAS scores at post-operation 1 h, 12 h, 24 h point, lower cortisol, higher 5-HT and hypocretin in serum than the placebo group.

**Conclusion:**

In this randomized clinical trial, dexmedetomidine can improve the sleep quality of patients undergoing endoscopic sinus surgery. These results suggest that this therapy may be a viable strategy to enhance postoperative sleep quality in patients with endoscopic sinus surgery.

**Trial registration:**

The study was approved by the Bethune International Peace Hospital Ethics Committee (2021-KY-129) and registered in the Chinese Clinical Trial Registry (ChiCTR2100051598, 28/09/2021).

## Background

As one of the important components of higher life activities, sleep is also a pointer to the health state of the body [1, 2]. When the body experiences sleep, the metabolism of the brain and body decreases. After sleep, the fatigue of the body disappears and the function is restored, which plays a pivotal role in maintaining normal body functions [3, 4]. Postoperative sleep disorders manifest sleep rhythm disorder or sleep structure and phase change, which occurred about 42% [5]. In particular, for patients who have undergone the major stimulation of surgery, adequate sleep plays a good role in promoting postoperative rehabilitation [6]. However, perioperative patients are affected by a variety of factors including inflammation, pain, nausea and vomiting, and their sleep is disturbed to vary degrees. Some patients also show sleep disorders, sleep deprivation, or even immune system disorders, which increase the risk of postoperative infection and the probability of complications and delay the recovery time of the body. It even aggravates the risk of comorbidities in patients' own cardiovascular and endocrine systems and even increases the perioperative mortality of patients [7]. It has been reported that 5-hydroxytryptamine (5-HT) and lateral hypocretin (also known as orexins) play important roles in the regulation of sleep and awakening [8–10].

Endoscopic nasal sinus surgery is a nasal and sinus surgery carried out by endoscopy, especially it can directly insight into the deep nasal cavity and sinus, and is suitable for cleaning the lesions in deep depressions and fissures, to help restore the ventilation and drainage function of the nasal and sinus [11]. However, endoscopic nasal sinus surgery is often associated with postoperative pain and postoperative sleep disorder [12], then aggravate the severity of postoperative pain and slow the recovery of patients [13, 14].

Dexmedetomidine (Dex), as a highly selective α2 adrenergic receptor agonist, has inhibition on postoperative immunosuppression and implementation on a sedative, analgesic, and anti-anxiety effects by acting on the peripheral and central nervous system [15]. Dexmedetomidine induces deep sedation simulation N3 sleep EEG patterns mimicking natural sleep [16] and could increase non-REM 2 sleep, decreased REM sleep [17]. In vivo, dexmedetomidine can effectively alleviate postoperative hippocampal inflammation, improve cognitive function or increase splenic TFF2 expression in sleep deprivation rats [18, 19]. Dexmedetomidine by gastrointestinal administration in mice could induce sedative and hypnotic effects by exciting the sleep-promoting nucleus and inhibiting the wake-promoting areas [20].

For endoscopic sinus surgery, dexmedetomidine is beneficial in providing good visibility during functional and decreasing intra-operative bleeding [21]. In this clinical trial, patients undergoing endoscopic nasal sinus surgery were enrolled to observe the effects of dexmedetomidine on postoperative sleep quality.

## Materials and methods

### Participants

This single-center randomized, double-blinded, controlled trial was approved by the Bethune International Peace Hospital Ethics Committee (Ethics No. 2021-KY-129) and registered in the Chinese Clinical Trial Registry (ChiCTR2100051598, 28/09/2021), was following the CONSORT guidelines. Then related risks were explained to patients and their families, consent of patients' families was obtained and informed consent was signed. This trial was initiated from November 1, 2021, to January 31, 2022. The inclusion criteria for this clinical trial were as follows: patients who underwent endoscopic sinus surgery with ASA grade I-II, age 18–65 years, and body mass index (BMI) from 20 to 30 kg/m^2^. Exclusion criteria were as follows: Cerebral hemorrhage or stroke; Severe dysfunction of important organs such as the heart failure, and uremia; A history of depression or sleep disorders requiring sedatives, Antidepressants, or other medications; History of mental illness; Second-degree or third-degree heart blockage; Obstructive sleep apnea–hypopnea syndrome, Patients or family members refuse.

### Randomization and masking

Eligible participants were randomized in a 1:1 ratio to receive dexmedetomidine or saline placebo during surgery. The random sequence is a computer-generated random number based on the network security system. A nurse who was not involved in the study design loaded the study drugs into the same 50 ml syringe according to the different groupings, and the grouping information was contained in sequentially numbered sealed envelopes. Surgeons and other study participants, as well as the patients themselves, were blind to the groupings.

### Sample size estimation

The sample size was calculated based on the sleep efficiency index(SEI) and taken as the main evaluation index, according to the pilot study, the SEI at the night after surgery was 64.8 (2.6) in the control group and 66.4 (3.0) in dexmedetomidine group. Assuming a two-tailed alpha threshold of 0.05 and a power (1-beta) of 90% at a significance, 48 participants in each group were required. Taking into consideration a 10% withdrawal and loss for follow-up, we finally recruited 110 patients for this study.

### Anesthesia procedure

Fasting for 8 h before surgery, patients were opened the peripheral venous access after entering the operation room. Non-invasive blood pressure, electrocardiogram, SpO2, HR, and bispectral index (BIS) were monitored. For anesthesia preparation, dexmedetomidine (Yangzijiang Pharmaceutical Co., LTD.) was diluted into 4 ug ML^−1^ with 0.9% sodium chloride solution. Patients in dexmedetomidine group was given 0.5 ug•kg^−1^ intravenous pumping at standard weight for 10 min before the establishment of peripheral post-intravenous anesthesia induction and then adjusted to 0.2ug•kg^−1^•h^−1^ intravenous pumping until 30 min before the end of surgery. The control group pumps an equal dose of normal saline. The induction scheme is as follows: Lidocaine (Tianjin Jinyao Pharmaceutical Co., LTD.) 1 mg•kg^−1^, Cisatracurium (Dongying Pharmaceutical Co., LTD.) 0.15 mg•kg^−1^, propofol (Braun, Germany) 1.5–2.5 mg•kg^−1^, Sufentanil (Renfu Pharmaceutical) 0.3ug•kg^−1^. After induction, mechanical ventilation was performed through endotracellar intubation, and the partial pressure of carbon dioxide at the end of respiration (PetCO_2_) was monitored at 35–45 mmHg, 4–8 mg•kg^−1^• h^−1^ continuous intravenous pumping of propofol and 0.1–0.3ug•kg^−1^•min^−1^ continuous pumping of remifentanil to maintain anesthesia. Intermittent injection of cisatracurium maintained muscle relaxation and BIS values between 40 and 60. Urapidil and dopamine were used to maintain blood pressure at no less or more than 20% of the baseline blood pressure. Patients will drop out of the study when they appear severe arrhythmias or hemodynamic disturbances. When the operation was completed, all anesthesia drugs were stopped and the patient was sent to the anesthesia recovery room (PACU). The patient was fully awake and could perform actions such as raising his head and shaking hands as instructed by the doctor, and the tracheal tube was fully hauled out.

### Outcome measures

When patients were safely returned to the ward, the Philips polysomnography monitor (Alice PDx, Netherlands) was used to monitor their sleep of patients. The sleep efficiency index (SEI), arousal index (AI), and the ratio of rapid eye movement sleep to total sleep (REM) of the patients were recorded 1 day night before surgery (T1) and the night after surgery (T2). At the same time, Ramsay’s sedation score was performed 1, 12, and 24 h after surgery, with the following criteria: 1 point: anxiety, agitation; 2 points: accurate orientation, quiet to cooperate with the operation; 3. Responds only to commands. 4 points: quick response to strong sound stimulation and tapping eyebrow; 5 points: insensitive to strong sound stimulation and tapping eyebrow; 6 points: no response to strong sound stimulation and tapping eyebrow. VAS scores were evaluated at 1, 12, 24, and 48 h postoperatively, with the following criteria: mark at one point on a single 10-cm line–- “no pain” on the left end (0 cm) of the scale and the “worst pain” on the right end of the scale (10 cm). 5 ml of venous blood was extracted from the patients at 22:00 the night before surgery (T1) and 12 h after surgery (T2), and injected into sodium citrate anticoagulant vacuum collection vessels. Centrifugation was performed at 1500 RPM for 10 min, and the supernatant was removed to a new EP tube in a -80 °C refrigerator (Qingdao Haier, China) for later use. Cortisol, 5-HT, and hypocretin were detected by enzyme-linked immunosorbent assay (Elisa) using a spectrophotometer (BioTek, USA) to measure absorbance at 450 nm.

### Statistical analysis

SPSS software (Version 23.0. Chicago, IL, USA) was used for statistical analysis. The Kolmogorov–Smirnov test was used to evaluate the distribution of variables. Quantitative data were expressed as mean ± SD or median and interquartile range, classified data is expressed in percentage (%). To compare patient characteristics and operative data between the groups, independent *Z* Tests for continuous variables, and Fisher exact tests or χ2 tests for categorical variables were performed. The VAS scores and Ramsay Sedation Scores were evaluated by the Mann–Whitney test and χ2 test for categorical variables. The difference was statistically significant (*P* < 0.05).

## Results

### Study population

**Fig. 1:**
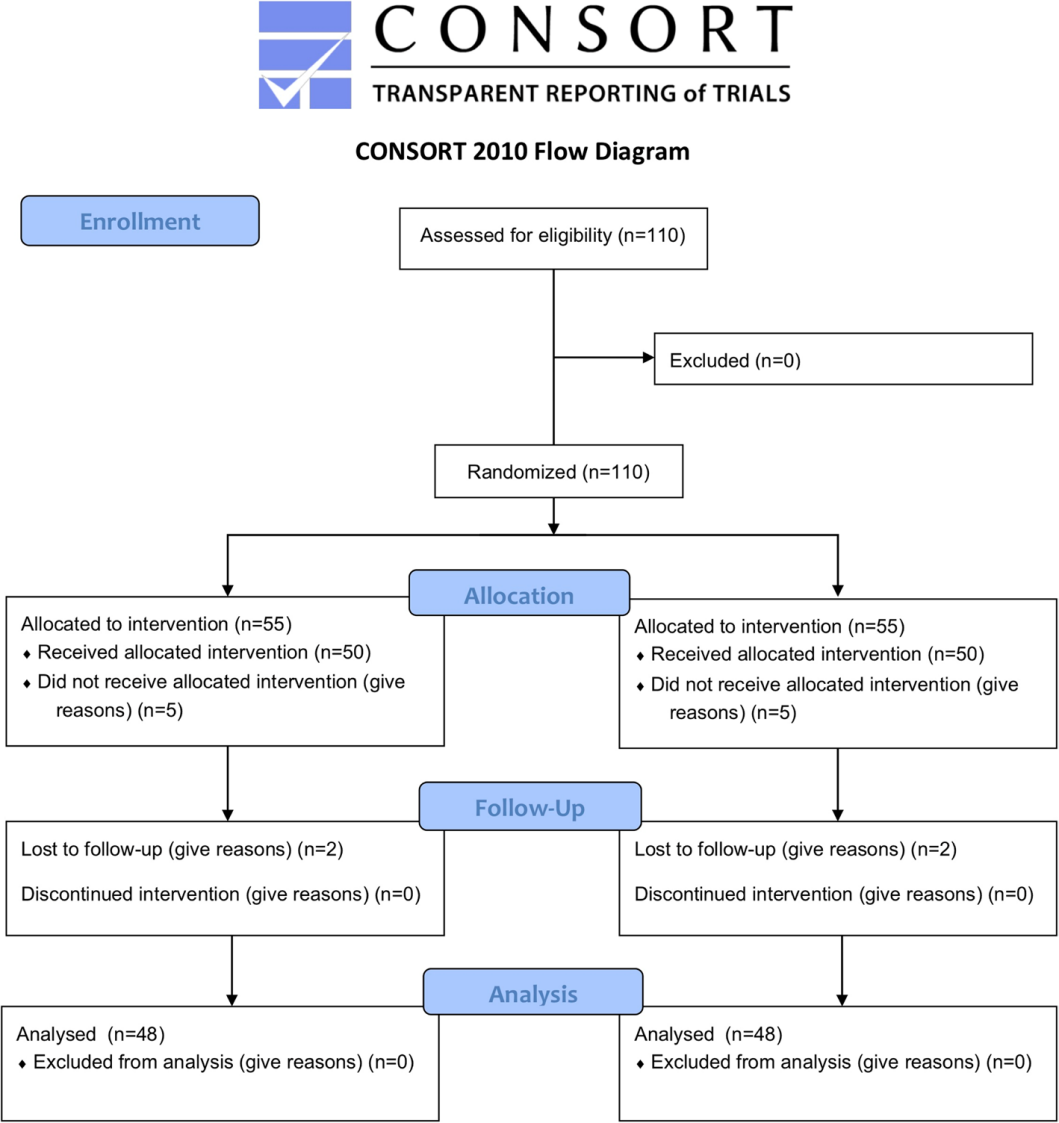
CONSORT 2010 flow diagram

A total of 110 eligible patients were excluded and randomized, 55 to dexmedetomidine and placebo (Fig. 1). 7 dexmedetomidine patients (3 surgical plans changed, 4 withdrew after surgery) and 7 patients in the control group (4 surgical plans changed, 3 withdrew after surgery). The remaining 96 patients (48 in each group) were included in the regimen-specific analysis. Baseline characteristics of the dexmedetomidine and control groups were similar age (mean, years, 41.52 [12.00] *vs* 44.83 [12.28]), American Society of Anesthesiologists status (eg, 31 patients [64.6%] *vs* 27 patients [56.3%] had class 2 status [indicating mild systemic disease]) (Table 1).

**Table 1:** Patient characteristics and intraoperative data

Characteristic	No. (%)		z Score or χ2 value	*P* value
	**Control group**	**Dexmedetomidine group**		
**Total patients, No**	48	48	NA	NA
**Age, mean (SD), y**	41.52(12.00)	44.83(12.28)	-1.228	0.219
**BMI, mean (SD)**	25.33(2.46)	24.82(2.46)	-1.016	0.310
**Sex**			1.053^c^	0.206
** Female**	19(39.6)	24(50.0)		
** Male**	29(60.4)	24(50.0)		
**ASA classification**			0.697^c^	0.266
** I**	17(35.4)	21(43.8)		
** II**	31(64.6)	27(56.2)		
**Time, mean (SD), min**				
** Surgical**	94.52(10.95)	93.54(10.36)	-0.224	0.823
** Anesthetic**	105.10(11.86)	104.38(10.96)	-0.088	0.930
** Smoking history(Y/N)**	20/28	23/25	0.379^c^	0.341
** Drinking history(Y/N)**	12/36	10/38	0.236^c^	0.404
** Sufentanil, mean (SD), μg**	29.90(5.00)	30.00(3.86)	-0.101	0.919
** Remifentanil, mean (SD), μg**	1112.83(143.85)	1074.10(175.28)	-1.327	0.185
** Propofol, mean (SD), mg**	686.48(77.46)	664.85(69.82)	-1.187	0.235
** Use of vasoactive drugs (%)**	8(16.7)	9(18.8)	0.071^c^	0.500
** Bleeding, median (IQR), ml**^**a**^	180(157.5–193.75)	180(155–190)	-0.132	0.895
** Transfusion volume, median (IQR), mL**^**b**^	950(862.5–1050)	1000(850–1050)	-0.445	0.656
** Urine output, median (IQR), mL**^**b**^	350(300–387.5)	342.86(300–400)	-0.396	0.692

*NA* Not applicable, *SD* Standard Deviation, *IQR* Interquartile Range, *Y* Yes, *N* No, *BMI* Body mass index, *ASA* American Society of Anesthesiologists

^a^Values were rounded to the nearest 5 mL

^b^Values were rounded to the nearest 50 mL

^c^χ2 value

### Primary outcome

**Fig. 2:**
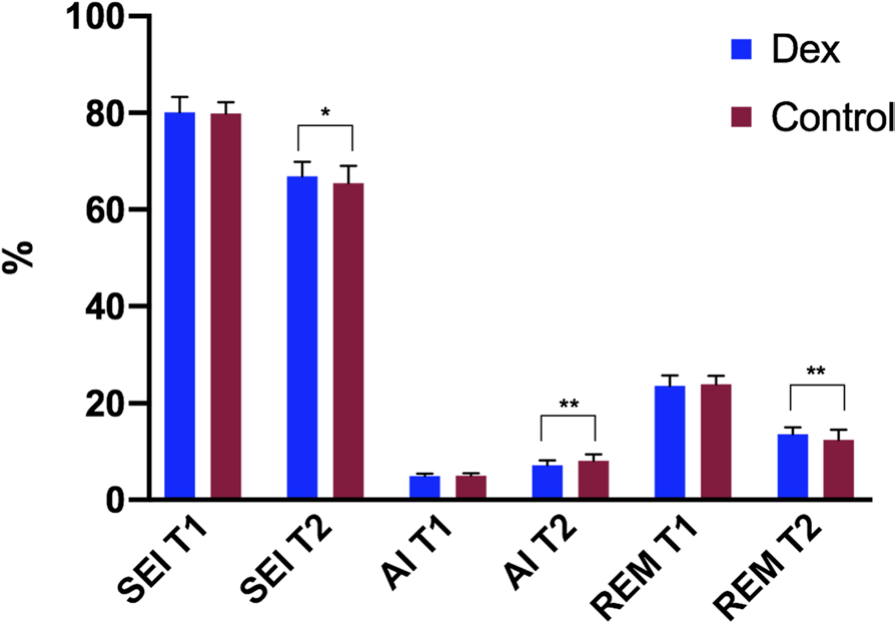
The primary outcomes at the two-point. At the T1 point without significant differences, T2 point, there were statistically significant differences in SEI, AI, and REM. Abbreviations: *SEI* Sleep efficiency index, *AI* arousal index, *REM* The ratio of rapid eye movement sleep to total sleep. *, *p* < 0.05, **, *p* < 0.01

The sleep efficiency index (SEI), arousal index (AI), and the ratio of rapid eye movement sleep to total sleep (REM) in T1 point were no significant differences between the two groups (*P* > 0.05). However, in the T2 point, there were statistically significant differences between the two groups in SEI, AI, and REM (*P* < 0.05, Fig. 2).

### Secondary outcomes

**Fig. 3:**
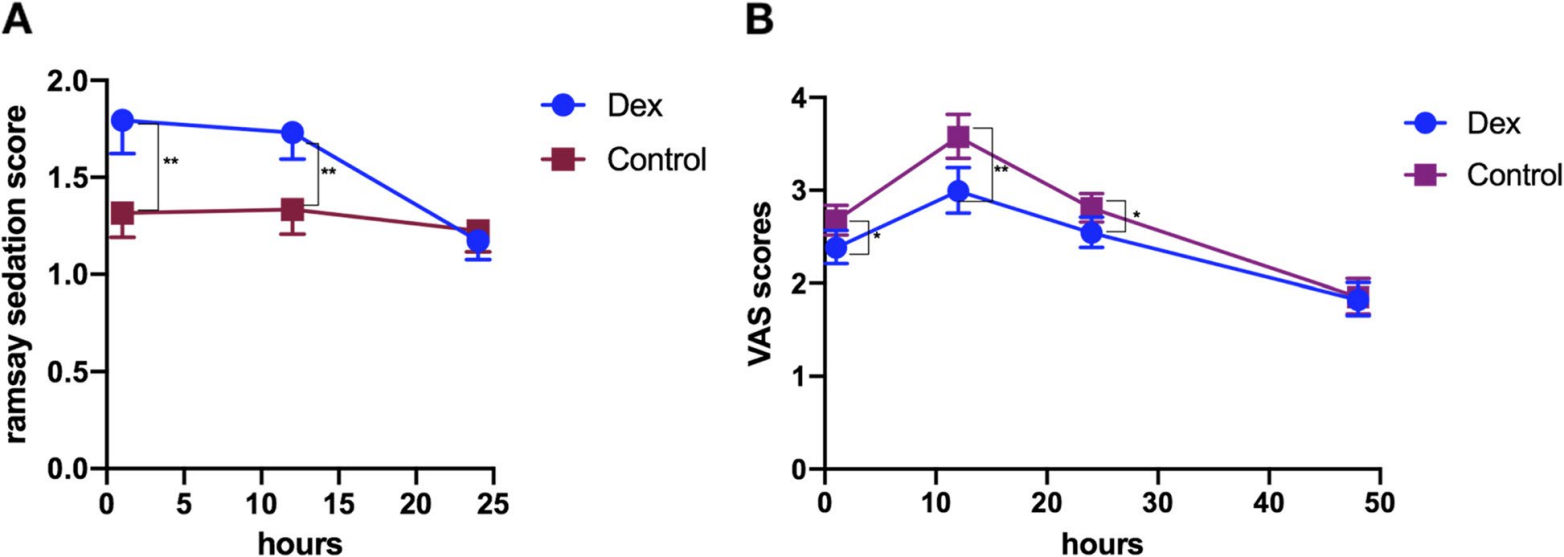
A The Ramsay sedation scores of the two groups with a statistical difference at 1 and 12 h after surgery, while with no statistical difference at 24 h. B The VAS scores showed statistical difference at 1, 12, 24 h after surgery, while no statistical difference at 48 h. *, *p* < 0.05, **, *p* < 0.01

**Fig. 4:**
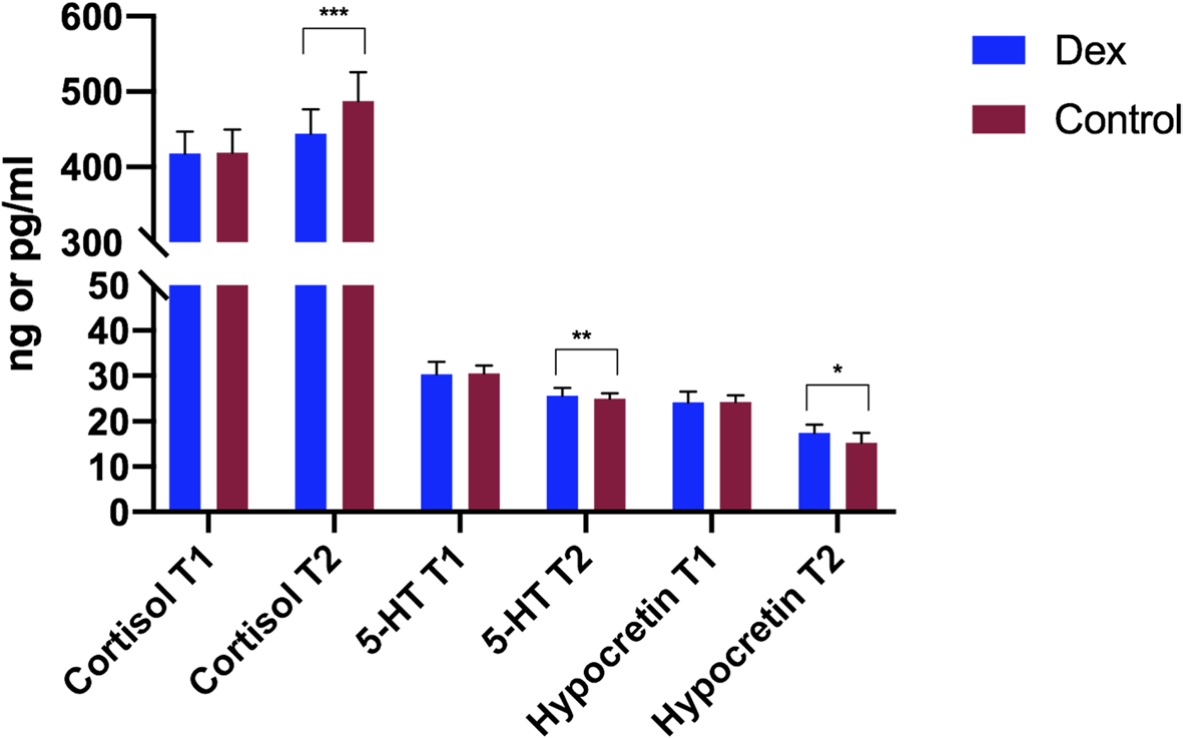
The cortisol, 5-HT, and hypocretin are at the two-point. There were no statistically significant differences at the T1 point. T2 point, the serum cortisol (ng/mL) of the dexmedetomidine group was lower, while the serum 5-HT (ng/mL) and hypocretin (pg/mL) were higher. Abbreviations: 5-HT, 5-hydroxytryptamine. *, *p* < 0.05, **, *p* < 0.01, ***, *p* < 0.001

Ramsay sedation scores of the two groups at 1, 12, and 24 h after surgery were compared, and there was a statistical difference at 1 and 12 h after surgery (*P* < 0.05, Fig. 3A), while there was no statistical difference at 24 h after surgery (*P* > 0.05). VAS scores showed statistical difference at 1, 12, 24 h after surgery (*P* < 0.05, Fig. 3B). There were no statistically significant differences in serum cortisol, 5-HT, and hypocretin at the T1 point (*P* > 0.05). However, at the T2 point, the serum cortisol of the patients in the Dexmedetomidine group was significantly lower than that of the Control group (*P* < 0.05), while the serum 5-HT and hypocretin of patients in the dexmedetomidine group were significantly higher than that of the Control group (*P* < 0.05), see Fig. 4, then the delirium and postoperative nausea and vomiting (PONV) at 24, 48 h were not significantly different but the PONV at 12 h was significantly lower (Table 2).

**Table 2:** The delirium and postoperative nausea and vomiting

Outcome	Control group	Dexmedetomidine group	χ2 value	*P* value
**Total patients, No**	48	48	NA	NA
**Delirium, No. (%)**				
12 h	4(8.3)	2(4.2)	0.711	0.339
24 h	3(6.3)	2(4.2)	0.211	0.500
48 h	0	0	NA	NA
**PONV, No. (%)**				
12 h	10(20.8)	3(6.25)	4.360	0.035
24 h	6(12.5)	2(4.2)	2.182	0.134
48 h	3(6.3)	1(2.1)	1.043	0.308

*NA* Not applicable, *PONV* Postoperative nausea and vomiting

## Discussion

Anesthesia is one of the factors affecting postoperative sleep-disordered [22, 23]. The incidence of postoperative sleep disorder in patients with general anesthesia and opioids is significantly higher than that in patients with regional anesthesia [24]. Postoperative pain is also one of the important factors leading to postoperative sleep disorders. Good perioperative analgesia can significantly improve postoperative sleep quality [25, 26]. Postoperative sleep disorders can lead to postoperative fatigue syndrome, paroxysmal hypoxemia, postoperative cardiovascular accidents, and mental state changes [27, 28]. Therefore, improving sleep quality has a profound impact on the recovery of patients undergoing surgery. Postoperative sleep disorders in patients undergoing nasal endoscopy can be induced by psychological changes, changes in breathing patterns, environment, postoperative pain, and throat irritation due to tracheal intubation. Regardless of the procedure’s complexity, most patients have varying degrees of postoperative sleep disorders [29]. Obesity patient and preoperative comorbid sleep apnea hypoventilation syndrome were positively associated with postoperative sleep disturbances [30, 31], and were excluded from our study in order to eliminate this influence.

The sleep efficiency index is the percentage of sleep time to bedtime, and the normal sleep efficiency is above 80%. If the sleep efficiency is less than 80%, it indicates that the patient has difficulty in maintaining sleep, and when it is less than 60% then suffering from serious sleep difficulty [32–34]. Increased rapid eye movement sleep improves sleep quality, with infants spending 50% of their sleep time in rapid eye movement sleep and adults nearly 20% [44]. The arousal index is the number of awakenings during sleep. The arousal index is usually less than 5 during normal sleep. An increase in this index indicates poorer sleep continuity and poorer sleep quality. The results of this study showed that the patients who experienced nasal endoscopy either sleep efficiency index decreased or arousal index increased obviously. Whereas dexmedetomidine could improve such sleep efficiency index or arousal index significantly, suggesting dexmedetomidine could improve patients’ sleep quality.

Natural sleep can be divided into two types, namely rapid eye movement (REM) sleep and non-rapid eye movement (NREM) sleep [35]. In REM sleep, the vital signs and metabolic rate of the body are similar to those in the awake stage, and lack of REM sleep can lead to decreased learning ability and even cognitive dysfunction [36]. REM sleep can represent a deep sleep state to a certain extent. Dexmedetomidine is a α2 receptor agonist that stimulates the α2 receptor in the locus coeruleus at the bottom of the fourth ventricle and the anterior dorsal pons to promote sleep [37]. Continuous infusion of dexmedetomidine can improve postoperative sleep quality by promoting patients to return to the normal circadian sleep cycle, improving sleep efficiency and structure [38–40]. In this clinical trial, the sleep quality of patient with dexmedetomidine during surgery was significantly improved with the SEI and proportion of REM elevated and AI descended 12 h after the surgery. Dexmedetomidine-induced sleep showed no significant difference from natural sleep in terms of sleep efficiency [41, 42]. The results of this study showed that when nasal endoscopy patients received dexmedetomidine intervention, the proportion of REM sleep was higher than that of the control group, suggesting that dexmedetomidine can increase the proportion of REM sleep in nasal endoscopy patients to improve postoperative sleep.

Trauma and surgical stress are the key factors affecting the quality of postoperative sleep, which has a direct effect on the central nervous system and changes the sleep architecture [43].Cortisol, widely known as the “stress hormone” [44]. 5-HT is an important neurotransmitter in the brain, widely distributed in the synapses and cerebral cortex, and can cause rapid eye movement sleep [45, 46]. When patients experience surgical stress with 5-HT decrease, the cerebral cortex activates and experiences sleep disturbances [47]. Hypocretin is a widely distributed in the hypothalamus area of neuropeptides, participates in the regulation of rapid eye movement sleep and the awakening of the body [48], increased the secretion can activate cortical cells of the body into the rapid eye movement sleep and plays a key role in promoting and maintaining wakefulness in mammals [49, 50]. The lack of hypocretin (orexin) may lead to narcolepsy in human beings. The results of this study suggested dexmedetomidine could reduce the level of cortisol, increase the secretion level of 5-HT and hypocretin to improve their postoperative sleep.

## Conclusion

Dexmedetomidine could improve the sleep efficiency index and increase the percentage of REM sleep time, reducing the postoperative arousal index, and this effect may be related to the decrease of serum corticosteroid levels and the increase of serum 5-HT and hypocretin in patients undergoing endoscopic sinus surgery. These results suggest that this therapy may be a viable strategy to enhance postoperative sleep quality while undergoing endoscopic sinus surgery.

## Limitations

There are still limitations in this study: (1) Postoperative sleep disturbance is closely related to the postoperative environment of patients, especially the surrounding sound and light stimulation, and these factors are not excluded in this study; (2) There are various factors that could lead to postoperative sleep quality, and we did not look for other related factors nor did we probe the mechanism of postoperative sleep quality. (3) We didn’t investigate the relationship between doses of dexmedetomidine and the improvement of postoperative sleep quality.

## Data Availability

The datasets used and analyzed during the current study are available from the corresponding author on reasonable request.

## Abbreviations

VAS: Visual Analog Scale
BMI: Body mass index
ASA: American Society of Anesthesiologists
SEI: Sleep efficiency index
AI: Arousal index
REM: The ratio of rapid eye movement sleep to total sleep
PONV: Postoperative nausea, and vomiting
5-HT: 5-Hydroxytryptamine
HR: Heart rate
PACU: Post-anesthesia recovery room
NREM: Non-rapid eye movement
